# Updating CMV protocols in lung transplant patients: a single-center case study modeling use of generative AI for antimicrobial stewardship protocol development and economic impact analysis

**DOI:** 10.1017/ice.2026.10480

**Published:** 2026-06-08

**Authors:** Kyle T. Enriquez, Augusto Dulanto Chiang, Casey Smiley, Milner Staub

**Affiliations:** 1 Medical Scientist Training Program, Vanderbilt University School of Medicine, Nashville, USA; 2 Division of Infectious Diseases, Department of Medicine, Vanderbilt University Medical Center, USA

## Abstract

**Objective:**

Antimicrobial Stewardship Programs (ASPs) need healthcare economic analyses to support and inform ASP strategies. This work aimed to determine whether widely available artificial intelligence (AI) platforms like Microsoft Copilot^TM^ could facilitate healthcare economics analyses for ASP programs without dedicated healthcare economic supports.

**Design:**

AI (Microsoft Copilot^TM^) was prompted to develop a cytomegalovirus prophylaxis protocol for lung transplant recipients using only PubMed-indexed articles. Copilot^TM^ was then prompted to produce probabilistic samples of simulated patients from aggregate statistics of a 165-patient cohort from Vanderbilt University Medical Center and to analyze cost-effectiveness across four distinct cytomegalovirus prophylaxis protocols, including its own.

**Setting:**

Tertiary care academic medical center, including outpatient and inpatient environments.

**Patients or participants:**

Simulated patient data was developed via random, single-blind, probabilistic selection from pre-defined aggregate cohort statistics.

**Results:**

The AI-generated prophylaxis protocol was evidence-based without hallucination, but this conservative protocol relied on outdated evidence and was associated with significant increases in expected per-patient cost (mean +$4740, *P* < .01) compared to recent guideline-based and institutional protocols. AI independently identified and executed sensitivity analyses, which revealed that in this simplified model, letermovir use had a large impact on expected per-patient cost.

**Conclusions:**

The AI-proposed protocol was less cost-effective, but data suggest that careful prompting can provide appropriate PubMed-indexed literature to support ASP protocol development. Additionally, CoPilot^TM^ provided a thorough cost-effectiveness analysis comparing all potential and existing protocols. With appropriate oversight, AI and Microsoft Copilot^TM^ can conduct healthcare economic analyses suitable for ASP strategic planning and implementation.

## Introduction

Antimicrobial stewardship programs (ASP) are vital to diagnosis and treatment of infectious diseases. These programs reduce healthcare costs, combat antimicrobial resistance, and support evidence-based care.^1–6^ To improve care in an equitable and sustainable fashion, ASPs must rigorously evaluate and report on outcomes from specific practices and protocols. A common challenge operational ASP teams face is assessing potential costs associated with planned interventions.^3,7,8^

Health economic expertise has contributed significantly to ASP at regional or national centers in under-resourced settings.^3,7,8^ However, local ASP operational leaders frequently have limited training or access to health economics expertise, despite their value in informing programmatic choices.^4,9,10^ Widely accessible tools to evaluate ASP interventions, including concrete cost-effectiveness comparisons to inform investment of resources into AS interventions, are needed.

Artificial Intelligence (AI) may address this gap in knowledge and facilitate development of cost-effective, evidence-based ASP strategies. Large language models (LLMs) have been applied broadly in healthcare where they function as accurate, adaptable tools to synthesize information from multiple sources and produce interpretable conclusions.^11–14^ AI platforms like Microsoft Copilot^TM^ allow for utilization of HIPAA-compliant, widely available, clinically applicable LLMs in healthcare.^11,12,15^ Appropriate applications of AI and LLMs to ASP work are currently underexplored.

CMV is a herpesvirus that can infect and recur in immunocompromised hosts, which manifests in detectable CMV DNAemia resulting in treatment (“CMV-DRT”).^17–19^ CMV-DRT can result in medication changes and increase the potential for medication side effects, acute rejection, and graft failure.^19^ The Vanderbilt University Medical Center (VUMC) ASP, Transplant Infectious Diseases (ID), and Lung Transplant Medicine faculty partnered to update institutional primary cytomegalovirus (CMV) prophylaxis protocols in lung transplant recipients. Prior retrospective chart review and algorithm-derived demographic data were used to define incidence and severity of CMV-related posttransplant outcomes at VUMC. These results, combined with new international guidelines for post-transplant CMV detection, prophylaxis, and treatment, led to a proposed new CMV primary prophylaxis institutional protocol.^16–18^

This work tested the efficacy of AI, via Microsoft Copilot^TM^, in generating an evidence-based CMV protocol, generating simulated patient cohorts from retrospective lung transplant cohort data, and estimating cost differences between protocols using these simulated patient samples. In addition, analyses were conducted in an investigator-initiated and AI-initiated manner to outline rates of protocol implementation and adoption to achieve projected returns on investment, adding to the comprehensive set of analyses that can be used to inform operational intervention decisions.

## Methods

### Acknowledgement of AI use

Given its wide availability and accessibility, we intentionally chose to utilize Microsoft Copilot^TM^, which is an LLM developed by OpenAI (Microsoft Copilot^TM^ v.1.25054.80.0, released in June 2025), to support model development and data analysis for this study. Copilot^TM^ was accessed through the VUMC Microsoft Teams subscription, as our center maintains a specific healthcare institutional license, and was used without modification. AI tools were employed only in areas explicitly labeled or indicated within the manuscript. At no point did AI engines or models have access to data protected under the Health Insurance Portability and Accountability Act (HIPAA).

### Protocol management and development

Protocols for CMV prophylaxis and management in lung transplant recipients were split across serostatus risk categories: low-risk Donor Negative (D-) and Recipient Negative (R-), intermediate-risk D-/R+ and D+/R+, and high-risk D+/R- or patients who underwent alemtuzumab induction. VUMC protocols pre- and post-2025 update were derived from transplant medicine and ID clinical expertise, with internal mapping of CMV-DRT lung transplant patient outcomes on pre-2025 protocol informing the post-2025 update.^16^ “International Guidelines” (IG) referred to the 4^th^ International Consensus Guidelines on the Management of Cytomegalovirus in Solid Organ Transplantation, while the protocol developed with Microsoft Copilot^TM^ was referred to as “AI-generated.”^18^ AI-protocolization required citation of PubMed-indexed articles which were manually confirmed (by *KTE*) as complete citations. Reference data used for the creation of AI-generated protocols were not manually updated and prompts were engineered using published best practices.^20,21^ Thorough documentation of each analysis step was conducted to allow for maximum transparency and reproducibility. Prompting and exact responses (Supp. Info. 1 and 2) and results (Table 1) were recorded.

**Table 1.** Generative AI via Microsoft Teams produces evidence-based but inappropriately conservative recommendations for protocol development within ASP teams Table 1 long description.

			VUMC (Prior to December 2025)		CMV International Guidelines^18^		VUMC (Post-December 2025)		AI-generated	
Risk for CMV reactivation	Donor	Recipient	*Treatment*	*Length of Prophylaxis*	*Treatment*	*Length of Prophylaxis*	*Treatment*	*Length of Prophylaxis*	*Treatment*	*Length of Prophylaxis*
Low	D-	R-	Valacyclovir 500 mg BID	3 mo	Valacyclovir	3 mo	Valacyclovir 500 mg BID	3 mo	Acyclovir 400 mg BID or Valacyclovir 500 mg BID	12 mo
Intermediate	D-	R+	Valganciclovir 900 mg PO daily	6 mo	Valganciclovir	6-12 mo	Valganciclovir 900 mg PO daily	6 mo	Valganciclovir 900 mg PO BID ×14 days → 900 mg PO daily	12 mo
	D+	R+	Valganciclovir 900 mg PO daily	6 mo	Valganciclovir	6-12 mo	Valganciclovir 900 mg PO daily	6-9 mo	Valganciclovir 900 mg PO BID ×14 days → 900 mg PO daily	12 mo
High	D+	R-	Valganciclovir 900 mg PO daily	9 mo	Valganciclovir	12 mo	Valganciclovir 900 mg PO daily	12 mo	Valganciclovir 900 mg PO BID ×14 days → 900 mg PO daily	12 mo
	*with intolerance		Letermovir 480 mg PO daily + Valacyclovir 500 mg PO BID	*refer to serostatus*	Letermovir	*refer to serostatus*	Letermovir 480 mg PO daily + Valacyclovir 500 mg PO BID	*refer to serostatus*	Letermovir 240–480 mg PO daily or IV ganciclovir	12 mo
	*with Thymoglobulin induction		Valganciclovir 900 mg PO daily	9 mo			Valganciclovir 900 mg PO daily	12 mo	Valganciclovir 900 mg PO BID ×14 days → 900 mg PO daily	12 mo

Protocols for primary prophylaxis for CMV in patients who receive lung transplantation, including those from VUMC before and after the time of retrospective manual chart review and assembly of aggregate statistics, those informed by the 4^th^ International Consensus Guidelines on the Management of Cytomegalovirus in Solid Organ Transplantation (which lacked dosing information), and those generated by Microsoft Copilot^TM^ as described in Table 1.

### Healthcare system cost assessment

VUMC healthcare system costs were calculated in collaboration with VUMC Pharmacy Business Analytics, Vanderbilt Medical Laboratories, and VUMC Department of Finance—Decision Support teams. Outpatient medicine costs to the health center were derived from VUMC FY25 costs per medication at listed doses. Based on these costs and expected use patterns,^18^ cost was empirically calculated per unit and per month (Supp. Table 1). Treatment dose medication costs were calculated for two-week courses (Supp. Table 2), as this is the *shortest* treatment a patient could receive for CMV reactivation at our center. All medication-specific calculations assumed normal renal function. All weight-dependent calculations were completed using average body mass index of 27.51 from the predefined lung transplant patient cohort.^16^

Inpatient costs associated with CMV-DRT were collected from the VUMC patient cohort, from which 21/165 (12.7%) patients had qualified inpatient costs. This distribution of costs was used to determine the expected daily inpatient costs ($8937.40) and incorporated into the AI cost model (Supp. Inf. 2).

### AI-derived health economics comparisons

**Figure 1.**
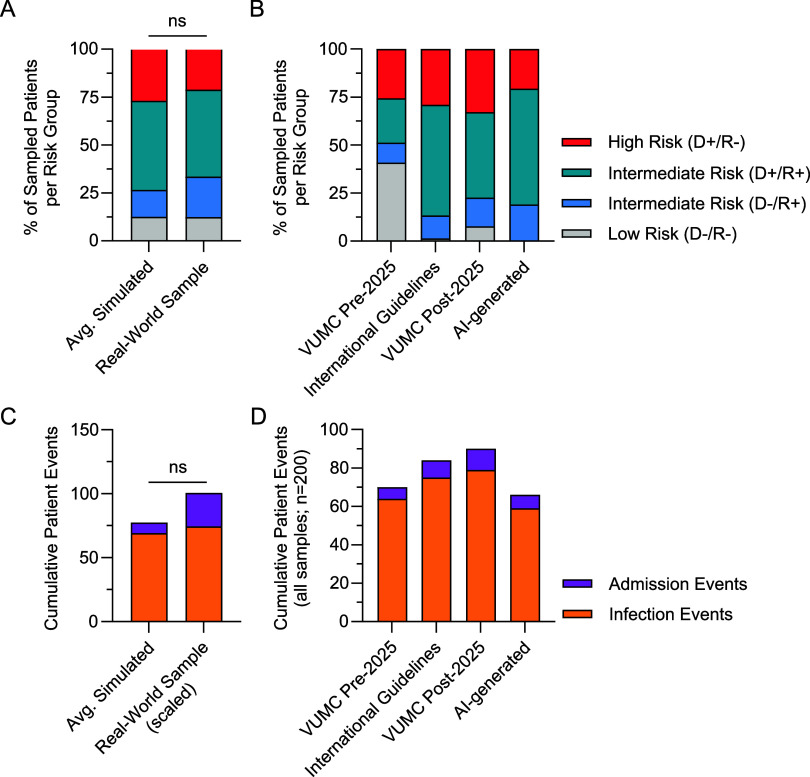
Figure 1 long description. **AI is an effective tool for sampling from a well-characterized patient population.** Evaluation of the proportion of patients across donor and recipient serostatus for (A) total and (B) individual protocols described in Table 1. Counts of cumulative patient events including CMV-DRT (Infection) and admission with a primary or secondary problem of CMV (Admission) across (C) total and (D) individual protocols described in Table 1. (A) evaluated by Wilcoxon matched-pairs signed rank test, while (B and D) evaluated by ordinary two-way ANOVA with main effects only. (C) evaluated by two-tailed ratio paired t-test, with all *P* > .05 = ns.

Microsoft Copilot^TM^ was also used to generate representative patient samples. Copilot^TM^ was provided aggregate data from the VUMC lung transplant cohort, including proportions of patients per serostatus grouping (12.42% D-/R-, 21.12% D-/R+, 45.34% D+/R+, and 23.6% D+/R-), proportion of patients who developed CMV-DRT in the 18 months following transplant (36%), and incidence of admission with a primary or secondary CMV diagnosis (12.7% without screening patients for premature death).^16^ Subsequently, Copilot^TM^ was prompted to calculate expected costs for each protocol presented in Figure 1. In this analysis, each protocol was applied to 10 random samples of 20 patients simulating a VUMC patient cohort.^16^ The size and number of these samples maintains appropriate statistical power at a level of α = 0.05, 1-β = 0.8, MDES = 1.5, assuming a two-tailed test and individual random assignment. Serostatus risk distribution, letermovir prescription rates, CMV-DRT and CMV-related admission incidence, etc. were predetermined from prior cohort studies at our center. Simulated patients were then assigned by CoPilot^TM^ to protocols in a single-blind, randomized fashion. Overall, simulated patients were required to reflect the original cohort described previously (Supp. Inf. 2).^16^ Fractional use data were calculated manually using a simple 10% stepwise implementation compared to the VUMC Pre-2025 protocol (Supp. Table 4).

### Statistical analysis

Statistical analyses and data visualizations were conducted with Microsoft Copilot^TM^ (Supp. Inf. 1 and 2), GraphPad Prism,^22^ Microsoft Excel,^23^ and Inkscape (v..92, https://inkscape.org/).

## Results

### AI-generated protocols for primary CMV prophylaxis in lung transplant recipients are overly conservative but literature-based

Detailed prompting (Supp. Inf. 1) successfully resulted in an AI-generated CMV prophylaxis protocol for lung transplant recipients (Table 1). The literature used by AI to support protocol generation was evidence-based with publication dates spanning 2012–2024.^24–32^ Copilot^TM^ successfully generated a strategy (Table 1) reflecting key clinical scenarios that influence patient outcomes, including transplant serostatus, medication intolerances, and high-risk induction immunosuppression.^18,33,34^

The AI-generated model was broadly more conservative with prophylaxis recommendations. Consistent with international guidelines^17,18^ and with retrospective VUMC cohort data,^16^ VUMC post-2025 CMV prophylaxis protocol extended primary prophylaxis in D+/R+ (intermediate risk) and D+/R- (high risk) patients to 9 and 12 months, respectively. Aligned with clinical practice recommendations, D-/R- (low risk) patients did not receive CMV prophylaxis in AI-generated protocols. However, AI-generated protocols instead suggested 12 months of acyclovir or valacyclovir to prevent herpes simplex or varicella zoster virus infection, compared to 3 months elsewhere (Table 1).^17,18^ Further, the AI-generated protocol suggested 12 months of CMV prophylaxis for both intermediate- and high-risk patients, with drug dosing changing between groups. The AI-generated protocol included a 2-week long induction dose approach for initiating prophylaxis, which was not described in other protocols (Table 1).

### AI can generate sample populations from aggregated cohort data to evaluate key cost drivers

From aggregate lung transplant cohort data, Copilot^TM^ successfully produced 10 probabilistic samples of 20 simulated patients and generated consolidated simulated-patient-level data, comparative analysis, and summary statistics (Supp. Table 4) to demonstrate its successes in this task.

The representation of CMV serostatuses across simulated patient samples and the patient cohort either in aggregate (Figure 1A) or across applied protocol samples (Figure 1B) were not significantly different across protocols. Further, the scaled incidence of CMV-DRT and CMV-associated admissions across the different simulated per-protocol samples (Figure 1C and D) were not significantly different.

### AI application of prophylaxis protocols to sampled populations enables comparison of expected costs

**Figure 2.**
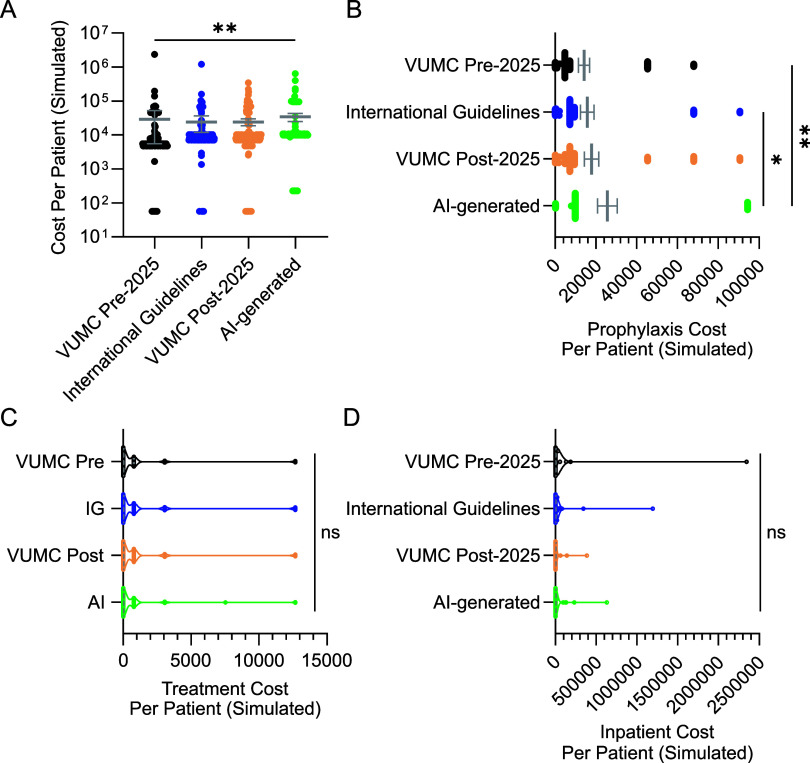
Figure 2 long description. **AI can sensitively measure and identify changes in expected cost associated with protocol use.** Assessment of (A) total cost per patient across 200 simulated patients within a shared fixed population, including the breakdown of cost across component categories (B) prophylaxis, (C) treatment, and (D) inpatient costs. (A and B) evaluated by Lognormal Brown-Forsythe and Welch ANOVA test with a posthoc Games-Howell multiple comparisons test, with individual variances computed for each comparison. (C and D) evaluated by Kruskal-Wallis test with posthoc Dunn’s tests, *P* > .05 = ns, *P* < .05 = *, *P* < .01 = **. Comparisons are not significant unless otherwise noted.

Prophylaxis, treatment, and inpatient costs were compared across protocols using the AI-generated samples, and among these comparisons only the AI-generated protocol resulted in significant total cost increases on a per-patient basis (Figure 2A). In this simplified model, sub-analyses identified this significant cost difference was driven by prophylaxis costs alone (Figure 2B) and not by observed differences in cost of treatment nor by inpatient cost (Figure 2C and D).

**Figure 3.**
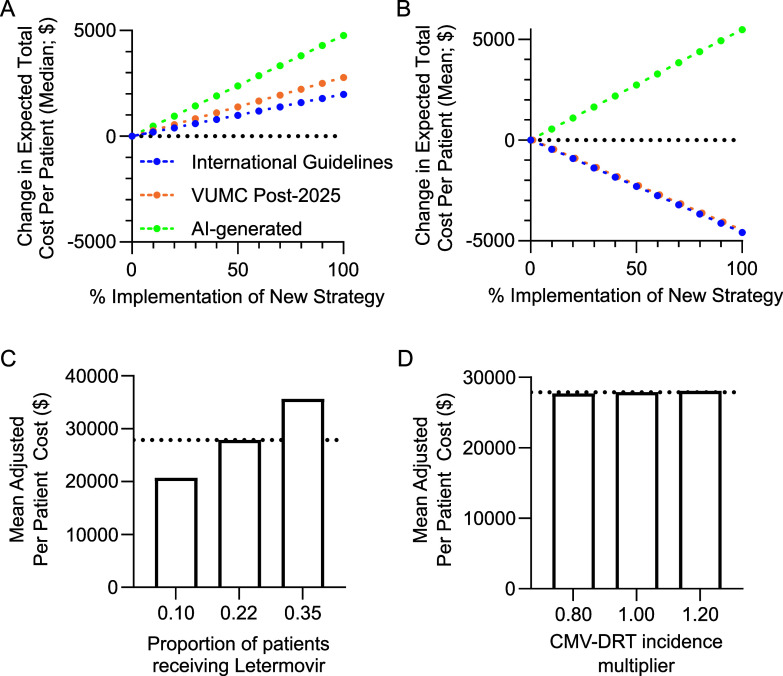
Figure 3 long description. **AI executes and proposes effective healthcare economics analyses to evaluate differences in protocols and potential implementation.** Balance measurements of fractional implementation of strategies vs. VUMC Pre-2025, specifically of evaluation of changes in (A) median and (B) mean total expected cost per patient. (C and D) describe AI-initiated sensitivity analyses including for changes in (C) proportion of patients that receive letermovir and (D) risk of patients developing CMV-DRT. In (C and D), dotted line indicates mean total expected cost per patient given aggregate statistics derived from manual chart review.

Copilot^TM^ successfully conducted a fractional implementation and sensitivity analysis. The distribution median and mean (Supp. Table 4) reflected increases in prophylaxis (lower cost, large proportion of patients) and inpatient cost (higher cost, low proportion of patients), respectively. Comparing all protocols (Table 1) to the VUMC Pre-2025 protocol (Figure 3A and B), implementation of the AI-generated protocol increased both median and mean total costs by >$5000/patient (Figure 3A and B). Implementation of either IG (Figure 3A and B; blue) or VUMC Post-2025 (Figure 3A and B; tan) protocols increased median cost per patient modestly ($1985 and $2779, respectively) but resulted in >$4500 decrease in mean per patient cost, despite no significant differences in cost associated with inpatient admission or treatment between protocols (Figure 2).

Serostatus was expected to influence CMV prophylaxis costs; however, beyond the provided prompts, Copilot^TM^ proposed and performed a sensitivity analysis evaluating the impact of proportion of patients on letermovir (Figure 3C and Supp. Inf. 2) and proportion with CMV-DRT (Figure 3D). This analysis showed that letermovir was associated with a ∼$7750 increase in expected per-patient cost (Figure 3C); however, simulating 20% higher CMV-DRT rates with fixed 2-week treatment courses did not significantly increase mean per patient cost (Figure 3D).

## Discussion

ASP teams are critical to the improvement of ID care globally.^1,6,7^ Lack of access to economic expertise is a barrier for ASP teams, as interventions that do not address cost and patient outcomes are less likely to be implemented.^3,8–10^ This work explored whether widely available and HIPAA-compliant AI tools like Microsoft Copilot^TM^ could assist ASPs in producing high-quality evidence-based protocols and if Copilot^TM^ could serve as a tool for economic analysis of health interventions, including addressing the question of variable impact depending on rate of adoption, a key driver in predicting ROI. We utilized updated CMV prophylaxis protocols in lung transplant recipients as a case study to answer these questions. This paper demonstrates that AI can use published evidence to generate a plausible clinical protocol and could utilize aggregate retrospective patient cohort statistics to simulate patient cohorts, generate simplified cost models, and predict care costs based on varying adoption of protocols, including predicted ROI depending on rate of successful adoption.

### AI is limited in generation of de novo clinical protocols

ASPs regularly evaluate clinical data to optimize and streamline infection prophylaxis and treatment protocols.^1,5,9,11^ Copilot^TM^ was successfully prompted (Supp. Inf. 1) to develop a CMV prophylaxis protocol for lung transplant recipients using PubMed-indexed references without AI “hallucinations,” which has been a key limitation in other settings.^37,38^ However, the AI-generated protocol was highly conservative, resulting in increased predicted healthcare system costs and extended patient drug exposure beyond evidence-based recommendations (Table 1). This may be associated with the time lag in clinical and operational expertise to be reflected in evidence-based literature for use within an AI-generated protocol. This finding supports the need for human clinical expertise in developing clinical protocols to balance side effects and infection risk, although AI was clearly useful in generating a workable prototype, potentially leading to less overall manpower utilized and faster turnaround times for protocol development.

### AI can generate meaningful, simple ROI analyses

Based on this documentation, AI effectively simulated patients from aggregate patient cohort data and validated that samples were representative of the larger cohort with regards to serostatus, infection rate, and admission rate (Figure 1). Further, CoPilot^TM^ generated logical models of the impact of each CMV protocol on these simulated, representative patient samples. The resulting models (Table 1 and Figure 2A and B) predicted increases in prophylaxis and total CMV-associated costs associated with newer protocols, consistent with prestudy expectations. AI was also able to effectively model how these different protocols would impact both expected mean and median cost per patient (Figure 3A and B), providing valuable information for ASP discussions with local leadership. The resulting models were able to place a price, based on our center’s costs, on each protocol’s impact with increasing adoption and appropriately demonstrated an increase in median cost per patient (expected with longer durations of prophylaxis) with an overall reduction in mean cost per patient (expected with less high-cost admissions).

The AI-generated protocol was shown to result in significantly higher mean and median costs per patient when compared to the IG, VUMC Pre-20205, and VUMC Post-2025 protocols. The VUMC Pre-2025 simulated cohort contained 40% low risk (D-/R-) patients who would not require CMV prophylaxis in all protocols and would instead receive valacyclovir for other viral prophylaxis, which per month is 40x less expensive than valganciclovir, potentially biasing cost assessment. However, as seen in Figure 2B, expected cost was significantly different between IG and AI-generated protocol costs despite a smaller proportion of low-risk patients. This supports the conclusion that the AI-generated protocol would result in higher expected costs. Further, Figure 2C and D demonstrated that there were no statistically significant differences in CMV-DRT treatment and inpatient costs between protocols. This was likely due to the small proportion of patients who experienced CMV-DRT and is consistent with real-world experience.

Further, new protocols are often adopted slowly, and benefits appear to be fractional.^35,36^ ASPs need to be able to present how differences in adoption can impact projected ROI, allowing for leadership planning and support in driving stakeholder change in practice. In this study, AI was able to generate compelling data (Figure 3A and B) to demonstrate the importance of supporting widespread adoption of the VUMC post-2025 protocol to maximize ROI. Taken together, the AI-generated protocol was likely reasonable in its clinical approach; however, it prioritized increased prophylaxis independent of thorough assessment of patient outcomes (CMV-DRT and inpatient admission).

### Validation of the margin of error for AI-generated models is needed

AI-generated simulated patient cohorts and associated costs were logical and consistent with real-world data. However, the translational potential of these models and AI as a tool to predict future costs and changes in patient outcomes is yet to be determined. Studies that perform these analyses using AI need to report on outcomes seen in healthcare systems to understand the margin of error that is present in these models and what factors, if any, form the minimal requirements for effective ROI modeling.

AI could potentially aid ASPs by providing additional analyses that may not have been considered, especially in programs without healthcare economic expertise. However, such analyses must be interpreted with caution. Independent of prompting, Copilot^TM^ conducted an informed sensitivity analysis (Supp. Inf. 2) choosing specific model features predicted to drive cost differences between groups and estimating the impact of adjusting their incidence. Figure 3C demonstrated the increased financial impact of increasing letermovir prescription. Figure 3D evaluated costs of CMV-DRT. While Figure 3D suggested that CMV-DRT may not be a significant cost burden, those costs did not include the 12.7% of patients that accrued additional inpatient care-associated costs. Therefore, Figure 3D only captured the minimal cost of medication-based treatment described in Supplemental Table 3, underscoring the need for ASPs to understand model components and publish prompts/data transparently in addition to validating generated simulations.

### Limitations of this work

The AI-proposed cost model generated was simple and could limit the direct application of this AI-ASP collaborative approach; however, this was chosen purposefully to illustrate the pros and cons of utilizing AI for healthcare cost modeling. AI was also prompted to produce simulated patient data from previous cohort statistics across protocols, which limits our interpretation of CMV-related outcomes. However, this increases the validity of a direct cost comparison between protocol groups and incorporates clinical data to inform future applications of this approach. Although likely not the ideal generative AI for healthcare economic analyses, the broad availability of Microsoft CoPilot^TM^ as an AI tool to analyze healthcare-derived data was modeled reliable performance and demonstrated wide accessibility. Future work would benefit from comparing across or between models or versions of models.

Outpatient testing was not included in health center costs. This was intentional as outpatient best practice is not standardized and could lead to confusion in the illustrative example.^16–18^ Treatment was assumed to have a fixed cost based on actual patient distributions (Supp. Inf. 2), but did not fully capture CMV-DRT cost variability. Letermovir switch for intolerance was captured in this model, but was independent of concurrent expected decrease in G-CSF cost because average length of G-CSF treatment was not characterized in the given patient cohort and could not be included. These examples highlight the limitations but also the flexibility and mounting complexity that can be attained utilizing AI models and the potential for iterative improvement. These data propose AI can use available data, agnostic of depth, and provide reasonable modeling of expected population and per-patient costs associated with implementation.

## Conclusion

Taken together, these data suggest generative AI (Microsoft Copilot^TM^) may be a valuable tool for evaluating healthcare economics questions in collaboration with ASP teams. The AI-generated protocol for primary CMV prophylaxis in lung transplant patients was not suitable for immediate implementation, supporting the need for human expertise when developing clinical protocols. However, AI was effective and accurate in generating representative simulated patient samples from a known patient cohort and executing economics analyses in an approachable format for operational AS leaders to generate data on potential costs of different strategies and suggesting helpful analyses beyond the initial prompting. Future work is needed to better understand the capacity for analysis and potential for failure when using AI to perform healthcare economic analyses.

## Supporting information

10.1017/ice.2026.10480.sm001 Enriquez et al. supplementary material 1 Enriquez et al. supplementary material

10.1017/ice.2026.10480.sm002 Enriquez et al. supplementary material 2 Enriquez et al. supplementary material

10.1017/ice.2026.10480.sm003 Enriquez et al. supplementary material 3 Enriquez et al. supplementary material

10.1017/ice.2026.10480.sm004 Enriquez et al. supplementary material 4 Enriquez et al. supplementary material

10.1017/ice.2026.10480.sm005 Enriquez et al. supplementary material 5 Enriquez et al. supplementary material

10.1017/ice.2026.10480.sm006 Enriquez et al. supplementary material 6 Enriquez et al. supplementary material

## Supplementary material

The supplementary material for this article can be found at https://doi.org/10.1017/ice.2026.10480.

## Figure 1. Long description

Bar graphs in (A) and (B) compare the percentage of sampled patients per risk group for different protocols. The x-axis represents different protocols, including Avg. Simulated, Real-World Sample, VUMC Pre-2025, International Guidelines, VUMC Post-2025, and AI-generated. The y-axis represents the percentage of sampled patients per risk group, ranging from 0 to 100 percentage. The graph is a stacked bar chart with four risk groups: High Risk (D+/R-), Intermediate Risk (D+/R+), Intermediate Risk (D-/R+), and Low Risk (D-/R-). Each bar is divided into segments representing these risk groups, with colors indicating different risk levels: red for High Risk, teal for Intermediate Risk (D+/R+), blue for Intermediate Risk (D-/R+), and gray for Low Risk. The graph shows the distribution of patients across these risk groups for each protocol. Bar graphs (C) and (D) compare cumulative patient events, including admission events and infection events, across different protocols. The x-axis represents different protocols, and the y-axis represents the cumulative patient events, ranging from 0 to 150. The graph is a grouped bar chart with two data series: Admission Events in purple and Infection Events in orange. Each group of bars represents a different protocol, showing the number of admission and infection events for each. The graph highlights the cumulative patient events for each protocol. All values are approximated.

Navigate back to Figure 1..

## Figure 2. Long description

The graph consists of four subplots labeled A, B, C, and D. Subplot A shows the total cost per patient across 200 simulated patients within a shared fixed population for different protocols: VUMC Pre-2025, International Guidelines, VUMC Post-2025, and AI-generated. The y-axis represents the cost per patient on a logarithmic scale, and the x-axis lists the different protocols. Subplot B breaks down the prophylaxis cost per patient, with the y-axis showing the cost and the x-axis listing the protocols. Subplot C shows the treatment cost per patient, with the y-axis representing the cost and the x-axis listing the protocols. Subplot D shows the inpatient cost per patient, with the y-axis representing the cost and the x-axis listing the protocols. The graph includes statistical annotations indicating significance levels with asterisks and ‘ns’ for non-significant comparisons.

Navigate back to Figure 2..

## Figure 3. Long description

The image contains four graphs analyzing healthcare economics. The first two graphs (A and B) are line graphs showing the change in expected total cost per patient (median in A, mean in B) as a percentage of implementation of new strategies compared to VUMC Pre-2025. Three strategies are compared: International Guidelines, VUMC Post-2025, and AI-generated. The x-axis represents the percentage implementation of the new strategy, and the y-axis represents the change in expected total cost per patient in dollars. The third graph (C) is a bar graph showing the mean adjusted cost per patient based on the proportion of patients receiving letermovir. The x-axis represents the proportion of patients, and the y-axis represents the mean adjusted cost in dollars. The fourth graph (D) is a bar graph showing the mean adjusted cost per patient based on the CMV-DRT incidence multiplier. The x-axis represents the CMV-DRT incidence multiplier, and the y-axis represents the mean adjusted cost in dollars. Dotted lines in graphs C and D indicate the mean total expected cost per patient given aggregate statistics derived from manual chart review. All values are approximated.

Navigate back to Figure 3..

## Table 1. Long description

Four different protocols for the primary prophylaxis of CMV in lung transplant recipients are presented. Each protocol is broken down into 4 risk sub groups by patient and donor CMV serostatus: low risk, or D-/R-; intermediate risk, either D-/R+ or D+/R+; or high risk, D+/R-. Each protocol is also split by the sources used to develop them. One protocol, used as the basis for comparison, is the VUMC protocol established based on practice guidelines before the start of this study in 2025. Other protocols are based on updated international consensus guidelines, on updated VUMC protocols after the initiation of this study in 2025, or on the careful prompting of Microsoft CoPilot (TM).

Navigate back to Table 1..
